# Oceanographic and biogeochemical drivers cause divergent trends in the nitrogen isoscape in a changing Arctic Ocean

**DOI:** 10.1007/s13280-021-01635-6

**Published:** 2021-10-09

**Authors:** Pearse James Buchanan, Alessandro Tagliabue, Camille de la Vega, Claire Mahaffey

**Affiliations:** 1 Department of Earth, Ocean and Ecological Sciences, University of Liverpool, 4 Brownlow Street, Liverpool, L693GP UK; 2 Leibniz Institute for Baltic Sea Research, Warnemünde, 18119 Rostock, Germany

**Keywords:** Biogeochemistry, Food webs, Primary production, Spatial ecology, Stable isotopes, Trophic position

## Abstract

Nitrogen stable isotopes (δ^15^N) are used to study food web and foraging dynamics due to the step-wise enrichment of tissues with increasing trophic level, but they rely on the isoscape baseline that varies markedly in the Arctic due to the interplay between Atlantic- and Pacific-origin waters. Using a hierarchy of simulations with a state-of-the-art ocean-biogeochemical model, we demonstrate that the canonical isotopic gradient of 2–3‰ between the Pacific and Atlantic sectors of the Arctic Ocean has grown to 3–4‰ and will continue to expand under a high emissions climate change scenario by the end of the twenty-first century. δ^15^N increases in the Pacific-influenced high Arctic due to increased primary production, while Atlantic sector decreases result from the integrated effects of Atlantic inflow and anthropogenic inputs. While these trends will complicate longitudinal food web studies using δ^15^N, they may aid those focussed on movement as the Arctic isoscape becomes more regionally distinct.

**Supplementary Information:**

The online version contains supplementary material available at 10.1007/s13280-021-01635-6.

## Introduction

The Arctic Ocean is experiencing the most rapid environmental and ecological changes on the planet. Surface air temperatures are rising by over twice the global average, which is causing the areal extent of summer sea ice to decline by roughly 13% per decade (Meredith et al. 2019). Warming and enhanced availability of light due to sea ice loss has stimulated phytoplankton growth and nutrient use, which has manifested as a 57% increase in marine primary productivity since 1998 (Lewis et al. 2020). At the same time, increased exchange with neighbouring Atlantic and Pacific Oceans (Spielhagen et al. 2011; Woodgate 2018) together with radiative warming is altering seasonal mixing (Polyakov et al. 2017) and will likely affect nutrient supply mechanisms (Henley et al. 2020), while also accelerating the invasion of boreal plankton (Oziel et al. 2020) and fishes (Fossheim et al. 2015) into the Arctic. There is little indication that these trends will reverse in the coming decades (Hinzman et al. 2013).

These multiple forcings are affecting Arctic food webs. Shifts in phytoplankton community composition, size structure and phenology (Li et al. 2009; Comeau et al. 2011; Mills et al. 2018; Oziel et al. 2020) are becoming evident (Ardyna and Arrigo 2020) and can initiate bottom-up cascades that affect higher order consumers (Bindoff et al. 2019). For example, a transition to smaller, less productive forms of phytoplankton (Li et al. 2009; Lee et al. 2013) may be accompanied by a transition to smaller species in higher trophic levels (Daufresne et al. 2009). Also, the success of large, lipid-rich copepods is strongly linked to the composition, timing and magnitude of the spring phytoplankton bloom (Feng et al. 2018) and as a primary food source for keystone fishes (Buren et al. 2014) can affect populations of predators such as harp seals (Stenson et al. 2016) and seabirds (Duffy-Anderson et al. 2019). Concurrent shifts in physical conditions can in turn alter the viable habitat of top predators and initiate top-down trophic cascades (Bindoff et al. 2019). For example, less polar bears (Laidre et al. 2008) and the incursion of killer whales into the Arctic as sea ice retreats (Breed et al. 2017) may affect the population and foraging behaviour of narwhal and other meso-predators to restructure food webs. Similarly, warming can accelerate parasitism and disease (Davidson et al. 2011), which alongside the invasion of boreal species threatens to severely alter the low-diversity, highly sensitive ecosystems of the Arctic (Callaghan et al. 2004).

In recognition of the sensitivity of Arctic ecosystems to accelerating anthropogenic influence, numerous studies have used stable isotopes as a means to construct and investigate the changing dynamics of Arctic food webs (e.g. Young and Ferguson 2014; Yurkowski et al. 2016, 2020; de la Vega et al. 2019). Stable nitrogen isotopes have featured prominently as a primary means to assess trophic level and diet. The biomass of higher trophic levels become enriched in the heavy isotope (^15^N) due to a preferential excretion of the lighter isotope (^14^N) following ingestion and amino acid processing. This leads to a step-wise increase in the ratio of ^15^N to ^14^N, measured in per mil (‰) as

$$\updelta^{15}\text{N}=\left(\frac{^{15}\text{N}/^{14}{\text{N}}{_{\text{sample}}}}{^{15}\text{N}/^{14}{\text{N}}{_{\text{standard}}}}-1\right)\cdot 1000,$$

that averages about 3 ± 1‰ per trophic level (Minagawa and Wada 1984; Post 2002). Consequently, the position of an organism in the food web, its diet, and how its position might respond temporally or spatially to environmental or ecological change can be garnered from δ^15^N.

An essential requirement of stable isotope investigations is knowledge of the “isoscape”, which is the baseline isotopic values of phytoplankton or detritus, at a scale relevant for the animal or food web of interest (Graham et al. 2010). Numerical models have been useful in this regard for predicting spatial and temporal patterns in the isoscape. For δ^15^N, ocean models have quantified variations between ocean basins on the order of 3‰ and thus equivalent to an entire trophic level (Somes et al. 2010; Buchanan et al. 2019). The Pacific Ocean has the highest δ^15^N values for nitrate and organic matter ($\updelta^{15} {\text{N}}_{{{\text{NO}}_{3} }}$ and δ^15^N_POM_), while the Atlantic has the lowest due to fundamental differences in basin-wide nitrogen cycling (Somes et al. 2010; Sigman and Fripiat 2019). This isotopic contrast manifests in the Arctic, where the presence of Pacific seawater in the Chukchi Sea, South Beaufort Sea, Canadian Archipelago, and along the Labrador shelf elevates $\updelta^{15} {\text{N}}_{{{\text{NO}}_{3} }}$ and δ^15^N_POM_ by 2–3‰ over the Atlantic-influenced Irminger, East Greenland and Barents Seas (de la Vega et al. 2021; Tuerena et al. 2021).

Alongside this oceanographic contrast, biogeochemical processes further modify $\updelta^{15} {\text{N}}_{{{\text{NO}}_{3} }}$ and δ^15^N_POM_ values. High rates of primary production and sedimentary denitrification along the path of Pacific inflow further raise already high $\updelta^{15} {\text{N}}_{{{\text{NO}}_{3} }}$ values (Granger et al. 2011). In contrast, the release of nitrogen from fossil fuel burning and fertiliser and its subsequent deposition in the Atlantic and European seas (Hauglustaine et al. 2014) could further decrease already low $\updelta^{15} {\text{N}}_{{{\text{NO}}_{3} }}$ values (Yang and Gruber 2016), such as is observed in the northwest Pacific (Ren et al. 2017). Local or remote biogeochemical processing of $\updelta^{15} {\text{N}}_{{{\text{NO}}_{3} }}$ has the potential to alter the isotopic gradients between the Pacific- and Atlantic-sector seas. Without a clear understanding of how and why the Arctic isoscape changes in space and time, it becomes challenging to not only construct food webs at Arctic-relevant scales but also to disentangle shifts in food web structure from shifts in oceanography and biogeochemical cycling (de la Vega et al. 2021). Robust management of Arctic ecosystems, therefore, requires knowledge of the isoscape and how it is changing.

In this study, we explore how and why the Arctic isoscape of nitrogen responds to rapid changes in environmental conditions using a global ocean-biogeochemical model (Aumont et al. 2015). We use both reanalysis-driven and emissions-driven simulations to investigate historical, contemporary and future changes in the Arctic nitrogen isoscape and their drivers. Using this suite of experiments, we identify a consistent anthropogenic influence that drives an intensifying isotopic gradient between the Pacific- and the Atlantic-sector seas.

## Methods

### Ocean model and nitrogen isotopes

We used the Pelagic Interactions Scheme for Carbon and Ecosystem Studies version 2 (PISCESv2) biogeochemical model, attached to the Nucleus for European Modelling of the Ocean version 4.0 (NEMOv4) general ocean circulation model (Aumont et al. 2015). The ecosystem component of the biogeochemical model includes two phytoplankton types (nanophytoplankton and diatoms), two zooplankton types (microzooplankton and mesozooplankton), small and large sinking particulate organic matter (POM), dissolved organic matter, oxygen, the full carbon system, water column and sedimentary denitrification, nitrification, annamox and an implicit nitrogen fixer group. This global ocean-biogeochemical model resolves oceanographic exchanges between the Arctic and the major oceans, accounts for climate-driven shifts in physical properties like sea ice extent and surface temperature, and predicts the resulting changes in biogeochemical properties, like nutrient concentrations, primary production and isoscape values. Horizontal model resolution varied between 0.5° at the equator, 2° in the subtropics and 1° poleward of 60°, while vertical resolution varied between 10 and 500 m thickness over 31 levels. Note that under-ice blooms that are likely an important contribution to primary production in the Arctic are not considered (Arrigo et al. 2012).

Nitrogen isotopes were integrated within PISCESv2 for the purposes of this study and are fully described in Appendix S1 and evaluated in Appendix S2 (Figs. S1, S2, S3, S4). We provide a brief overview here. The nitrogen cycle of PISVESv2 includes two active tracers of nitrate (NO_3_) and ammonium (NH_4_) that are assimilated, remineralised and excreted by the ecosystem, are introduced via nitrogen fixation and nitrification, and are removed via burial, anammox or denitrification in the sediments and water column. Nitrogen and its isotopes are cycled through these inorganic nitrogen forms and integrated into the phytoplankton and zooplankton biomass pools, which together contribute to pools of particulate and dissolved organic matter via their mortality and waste products. POM is thus a detrital product that is either re-consumed by zooplankton or recycled and subsequently assimilated by phytoplankton. For the purposes of this study, we focus our analysis on the isotopic signature of the POM (δ^15^N_POM_) that is the integrated product of this low-level marine ecosystem model, and represents the base of marine food webs.

Processes of relevance for the Arctic Ocean nitrogen isoscape include changes in the contribution of Pacific and Atlantic water, phytoplankton assimilation of nitrogen (i.e. primary production), sedimentary denitrification, and external inputs of nitrogen from rivers and atmospheric deposition. Pacific water is elevated in δ^15^N by 2–3‰ relative to Atlantic water (Somes et al. 2010; Buchanan et al. 2019) and so changes in the relative contribution of Pacific or Atlantic water to water masses within the Arctic alters the isoscape. Phytoplankton assimilation and sedimentary denitrification both increase the δ^15^N of inorganic nitrogen, typically nitrate ($\updelta^{15} {\text{N}}_{{{\text{NO}}_{3} }}$), and by raising the values of $\updelta^{15} {\text{N}}_{{{\text{NO}}_{3} }}$ they also raise the δ^15^N_POM_ as phytoplankton assimilate nitrate into their cellular matter (Karsh et al. 2012). These processes tend to fractionate at roughly 5‰ and 3‰, respectively (Sigman and Fripiat 2019), and their rates are highly correlated to one another in our simulations. Due to their strong correlation, as well as the recently observed increases in primary production linked to greater nutrient assimilation in the Arctic (Lewis et al. 2020), we consider primary production as the major biogeochemical player for isoscape changes, but acknowledge that sedimentary denitrification makes an important contribution in some regions (Granger et al. 2011). Inputs of nitrogen from rivers and from the atmosphere are poorly constrained but carry low δ^15^N signatures. For our model we chose 2‰ and − 4‰ for the addition of nitrate from rivers and atmospheric deposition, respectively (Sigman and Fripiat 2019).

### Simulations

We first achieved equilibrated solutions of biogeochemical tracers (i.e. nutrients, phytoplankton biomass, isotopic signatures) to initialise our experiments. To do so we ran the global ocean-biogeochemical model for 5000 years following the introduction of nitrogen isotopes within the model. This spin-up simulation occurred under constant preindustrial boundary conditions of 284 ppm CO_2_, preindustrial nitrogen deposition rates and with a repeating annual circulation field representative of the contemporary ocean state.

Our experiments were initialised from the three-dimensional output of this spin-up simulation and were either (i) reanalysis-driven or (ii) emissions-driven. Reanalysis-driven simulations involved forcing our ocean-biogeochemical model with a “best-guess” reconstruction of global atmospheric conditions over recent decades. This simulation therefore attempted to reproduce variations and trends in ocean properties, including the isoscape, under a realistic historical climate. In contrast, emissions-driven simulations involved forcing our ocean-biogeochemical model with historical and future anthropogenic emissions, which emulated climate change but with trends and variability unique to the climate model. Hence, the emissions-driven simulations produced long-term anthropogenically induced climate change, but had rates of change and oscillations that were different from the real climate. The advantage of emissions-driven simulations is that they isolate the effect of increasing emissions, while reanalysis-driven simulations emulate the trends and variability of recent decades. If similar trends emerge in both simulations, this strongly suggests that anthropogenic emissions are the dominant driver.

Reanalysis-driven simulations followed the protocols of the Ocean Modelling Intercomparison Project (OMIP; Orr et al. 2017). The NEMO–PISCESv2 ocean model was forced by the Japanese atmospheric reanalysis over the years 1958 to 2019 using 3-hourly bulk fluxes (Tsujino et al. 2018). Six repeat cycles of this 62-year forcing (372 years) as recommended (Tsujino et al. 2020) were made beginning on the 1st January 1648 Common Era (ce), ending on 31st December 2019 ce. Thereafter, the beginning of each cycle was initialised with the end of the previous cycle. Only output in the final cycle was used in analysis, and due to unavoidable initialisation of the final cycle with the end of the fifth cycle, the first 12 years of the final cycle (1958–1969 ce) were not included in any analysis to eliminate anomalous trends. We therefore focus on 1970–2019 ce for the reanalysis-driven simulations.

Emissions-driven simulations involved forcing the NEMO–PISCESv2 ocean-biogeochemical model with physical conditions provided by the IPSL-CM5A-LR Earth System Model under historical conditions until 2005 ce and following the Representative Concentration Pathway 8.5 (RCP8.5) between 2005–2100 ce (Riahi et al. 2011) as part of the Coupled Model Intercomparison Project phase five (Dufresne et al. 2013). The RCP8.5 scenario is commonly referred to as the “business as usual” scenario and involves continued high levels of greenhouse gas emissions through to end of century. This scenario was chosen to explore the greatest rate and magnitude of change. In addition, a preindustrial control simulation where no anthropogenic emissions occurred was also conducted alongside the emission-driven scenario. The preindustrial simulation included variability associated with only solar insolation cycles and internal modes of variability. The emissions-driven simulation included both natural variability as well as the historical and future conditions associated with RCP8.5 (Riahi et al. 2011).

While both sets of simulations involved changes to freshwater fluxes from rivers as part of the boundary conditions, we did not consider changes in nutrient fluxes from Arctic rivers (Terhaar et al. 2021) in this study.

### Nitrogen deposition

Aeolian reactive nitrogen (N_r_) deposition evolved over the simulations according to historical measurements and reconstructions. Prior to 1851 ce, N_r_ deposition was held at preindustrial levels (11 Tg N year^−1^). Onwards from 1851 ce, aeolian N_r_ deposition was increased using fields of Hauglustaine et al. (2014) that account for the anthropogenic and natural changes. Linear interpolation was used on N_r_ deposition fields to estimate years in between those estimated by Hauglustaine et al. (2014), being 1850, 2000, 2030, 2050 and 2100 ce. However, in order to represent the amplification of N_r_ deposition since 1950 ce (Galloway 2014), 60% of the increase between 1850 and 2000 ce occurred from 1950 ce onwards.

### Multiple linear regression

Drivers of change in the Arctic nitrogen isoscape were identified by applying a multiple linear regression analysis on output from the reanalysis-driven simulation. Predictor variables were salinity, N* (defined as NO_3_ − 16 * PO_4_) (Gruber and Sarmiento 1997) and the concentration of nitrogen in POM. These variables were carefully chosen to maximise parsimony and their ability to provide independent insight. Salinity as a purely physical tracer largely reflects contributions from fresh Pacific and salty Atlantic water, but deviations from this rule occur due to non-negligible inputs from rivers, sea ice formation/melt and evaporation/precipitation. N* reflects contributions from N*-negative Pacific water and N*-positive Atlantic water and is therefore also a water mass tracer (Gruber and Sarmiento 1997). The formula for N* involves multiplying phosphate (PO_4_) by 16 because for every atom of phosphorus within phytoplankton organic matter there is on average 16 atoms of nitrogen (Redfield 1958). Deviation from zero means that nitrogen is either in excess (positive N*) or depleted (negative N*) relative to the amount of phosphorus that is available to phytoplankton for their growth, and these deviations are driven by regional imbalances in the sources (e.g. atmospheric N_r_ deposition) and sinks of nitrogen (Gruber and Sarmiento 1997). Finally, the concentration of particulate organic nitrogen reflects the influence of biogeochemical transformations on the isoscape that elevate δ^15^N, namely phytoplankton assimilation during primary production in this study. Overall, this meant there were a total of eight potential statistical models, including the null model with no predictor (Table 1).

**Table 1:** List of statistical models used in the multiple linear regression analysis to discern the major drivers of δ^15^N_POM_ in the Arctic. Salinity is measured in units of practical salinity units (psu). N* is in units of mmol m^−3^ and is an index of the relative amount of nitrate to phosphate in seawater (nitrate − 16 * phosphate) where phosphate concentrations are scaled by the average requirements of phytoplankton (Gruber and Sarmiento 1997). POM is in units of mmol m^−3^ and is particulate organic matter concentration. Both predictor and response variables were averaged over the upper 100 m of the ocean model

Statistical models (reanalysis-driven)	Statistical models (emissions-driven)
δ^15^N_POM_ ~ salinity + N* + POM	δ^15^N_POM_ ~ N* + POM
δ^15^N_POM_ ~ salinity + N*	δ^15^N_POM_ ~ N*
δ^15^N_POM_ ~ salinity + POM	δ^15^N_POM_ ~ POM
δ^15^N_POM_ ~ N* + POM	δ^15^N_POM_ ~ 1
δ^15^N_POM_ ~ salinity	
δ^15^N_POM_ ~ N*	
δ^15^N_POM_ ~ POM	
δ^15^N_POM_ ~ 1	

Predictors (salinity, N*, POM) and δ^15^N_POM_ as the response variable were averaged annually and over the upper 100 m, such that only inter-annual changes with relevance for the surface ocean were included. Only grid cells north of 50°N were included in this analysis. All predictors were standardised (scaled to mean = 0 and standard deviation = 1) to facilitate the comparison of their effect size. We excluded from further analysis any grid cells for which predictors were significantly colinear (variance inflation factors > 3.0). Model selection was based on the information-theoretic approach through Akaike’s information criterion scaled for small sample sizes (AIC_c_). We compared a list of meaningful candidate models at every grid cell, with the maximal model being δ^15^N_POM_ = salinity + N* + POM. For each specific model, we calculated the AIC_c_, the difference between AIC_c_ and the best model (ΔAIC_c_), and the AIC_c_ weight (normalised weight of evidence in favour of the specific model, relative to the whole set of candidates). If all models had a higher AIC_c_ than the null model (δ^15^N_POM_ = 1), then the effects of all predictor variables were deemed non-significant. When multiple models had lower AIC_c_ than the null model and had an ΔAIC_c_ < 4.0, we used model averaging to produce averaged estimates of effect sizes and their 95% confidence intervals (Burnham and Anderson 2002). A predictor had a significant effect on δ^15^N_POM_ if its 95% confidence interval did not cross zero.

### Ocean model assessment

We undertook a thorough model-data assessment of both physical and biogeochemical properties using the reanalysis-driven simulations only (Appendix S2) due to their better representation of historical conditions. Model-data correlations of sea ice concentration and sea surface temperature indicated good agreement, although a model bias was that warming and melting of sea ice began too late in the season each year. Consequently, the seasonal cycle of primary production in the Arctic was delayed compared to the observations. Sea surface height was also assessed in the form of climate indices, namely the Subpolar Gyre Index in the North Atlantic (Koul et al. 2020) and the Arctic Oscillation in the central Arctic. Both showed good agreement with a reanalysis product heavily constrained by observations.

In addition to these physical comparisons, we also utilised a global compilation of $\updelta^{15} {\text{N}}_{{{\text{NO}}_{3} }}$ (Rafter et al. 2019) to assess the model performance in terms of its nitrogen isotope routines. This analysis revealed a moderate agreement with the in situ data and a slight negative bias in all values. However, in the context of other global biogeochemical models, the model-data fit was excellent (Buchanan et al. 2019). Importantly, the Atlantic–Pacific gradient in $\updelta^{15} {\text{N}}_{{{\text{NO}}_{3} }}$ was well resolved.

## Results

### Reanalysis simulations and historical changes

**Fig. 1:**
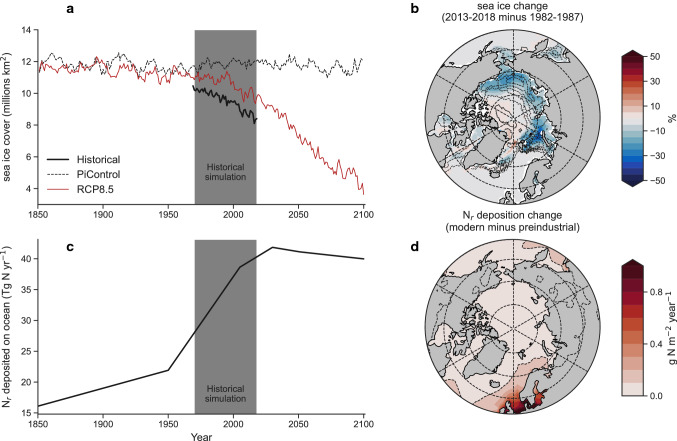
Major changes affecting the isoscape of nitrogen in the Arctic Ocean. **a** Time series of sea ice cover (millions km^2^) in our reanalysis-driven (solid black line) and emissions-driven (red line) simulations compared with preindustrial control conditions (dashed black line). **b** Observed spatial change in sea ice concentration 2013–2018 minus 1982–1987 (shading) and reanalysis-driven change (contours). Contours are in 5% intervals. **c** Time series of integrated change in aeolian reactive nitrogen (N_r_) deposited to the global ocean. **d** Change in N_r_ deposition between modern (2005) and preindustrial (1850) in the Arctic region. The dashed contour represents 0.1 g N m^−2^ year^−1^

**Fig. 2:**
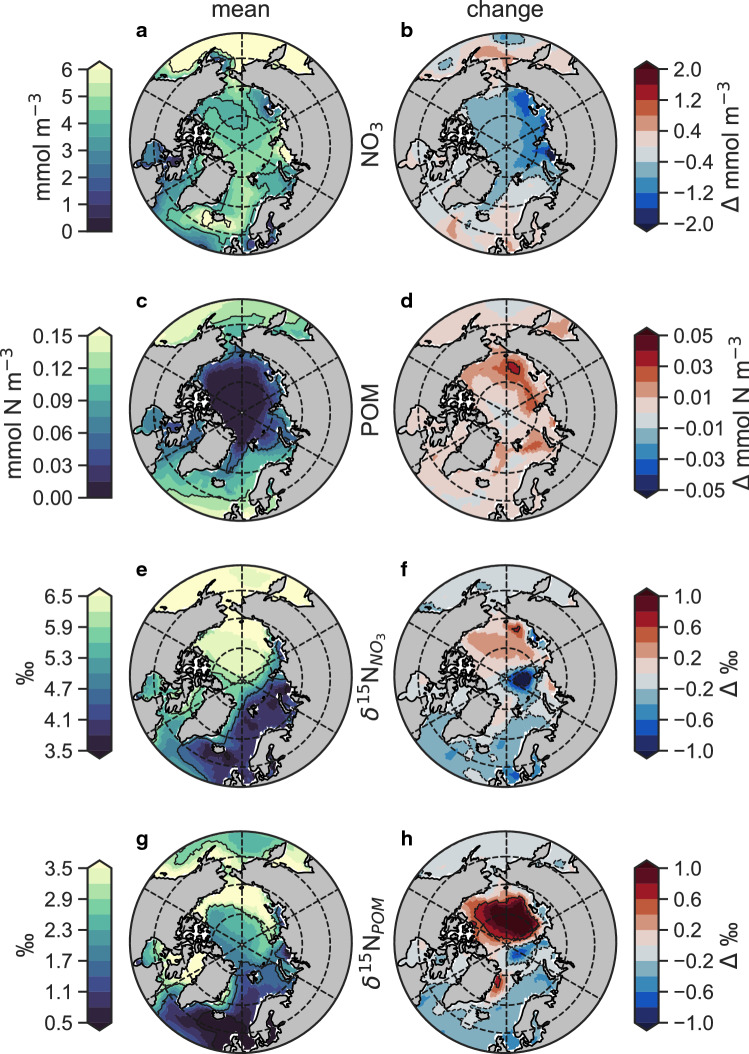
Annual mean conditions (1970–1990) and historical changes (2009–2019 minus 1970–1990) in surface properties and the nitrogen isoscape. **a** Concentration of nitrate (NO_3_) and **b** its change (*Δ*). **c** Concentration of particulate organic matter (POM) and **d** its change. **e** Values of δ^15^N (isoscape) of NO_3_ and **f** its change. **g** Values of δ^15^N (isoscape) of POM and **h** its change. All values come from the reanalysis-driven simulation of historical conditions

Major changes to the Arctic Ocean with relevance to the isoscape for our reanalysis simulation (1970–2019 ce) include a decline in sea ice extent (Fig. 1a, b), changes in Pacific and Atlantic seawater exchange (Fig. S5), and an increase in anthropogenic inputs of reactive nitrogen (N_r_) to the ocean (Fig. 1c, d; Hauglustaine et al. 2014). Warming and a decline in sea ice are important for the isoscape if phytoplankton production of organic matter increases, which would elevate δ^15^N values (Karsh et al. 2012; Sigman and Fripiat 2019). Our reanalysis-driven simulation showed good agreement with sea ice trends (Fig. 1b; Fig. S6; Spearman’s rank correlation = 0.97), albeit with a one month lag in autumn freezing and spring melting (RMSE = 1.04 million km^2^), and good agreement with increases in primary production observed over the past two decades across most regions of the Arctic Ocean (Lewis et al. 2020; Fig. 2d; Fig. S7). The increase in Pacific inflow since the 1990s (Woodgate 2018) was not reproduced, but a multi-decadal increase in Atlantic water inflow to the Barents Sea (Spielhagen et al. 2011) was evident in our simulation (Fig. S5). Finally, the rise in N_r_ deposition (Hauglustaine et al. 2014) with low δ^15^N values (Sigman and Fripiat 2019) directly affected the Atlantic-sector seas, namely the East Greenland and Barents. However, far-field N_r_ inputs further south at temperate and subtropical latitudes could also affect the isoscape if increasing Atlantic inflow carried more ^15^N-deplete N_r_ northwards. Note that while changes in glacial and fluvial freshwater fluxes were included (Tsujino et al. 2018), temporal changes in nutrient inputs from rivers and coastal erosion that stimulate primary production over Eurasian shelves (Terhaar et al. 2021) were not considered.

These physical changes resulted in a multi-decadal (mean conditions in 2009–2019 minus mean conditions in 1970–1990) drawdown of surface NO_3_, increases in organic matter, and an increase in the isotopic gradient between the Pacific-influenced seas of the high Arctic and the Atlantic-sector seas. Nitrate declined by 0.5–1 mmol m^−3^ over the central and eastern Arctic (Fig. 2a, b), and was coincident with increases in particulate organic matter symptomatic of increased primary production (Fig. 2c, d). The strongest response co-occurred with areas of sea ice loss, namely between 70°N and 80°N along the Southern Beaufort Sea, the Siberian shelves, the northern Barents Sea, and the west Greenland Sea. Meanwhile, weak declines or even slight increases in surface NO_3_ occurred in the Atlantic-sector seas of the Labrador, Barents, East Greenland and Irminger Basins, possibly symptomatic of increasing NO_3_ transport into the region from the North Atlantic (Spielhagen et al. 2011) and/or increased seasonal mixing associated with the erosion of salinity stratification (Polyakov et al. 2017). These seas also experienced declines in $\updelta^{15} {\text{N}}_{{{\text{NO}}_{3} }}$ and δ^15^N_POM_ (Fig. 2e–h). In contrast, the Pacific-influenced high Arctic and in particular the Beaufort Gyre experienced a strong increase in $\updelta^{15} {\text{N}}_{{{\text{NO}}_{3} }}$ and δ^15^N_POM_ coincident with NO_3_ drawdown and increases in organic matter concentrations. Consequently, the isotopic gradient between the Pacific and Atlantic sectors of the Arctic (Fig. 2e, g) increased from roughly 2‰ between 1970 and 1990 ce to 3‰ for $\updelta^{15} {\text{N}}_{{{\text{NO}}_{3} }}$ and to 4‰ for δ^15^N_POM_ by 2009–2019 ce (Fig. 2f, h). The magnitude of divergence between the Pacific and Atlantic sectors is consistent with recent field data reporting a $\updelta^{15} {\text{N}}_{{{\text{NO}}_{3} }}$ difference of 2‰ between the Barents Sea and the Canadian Archipelago using data from 2017 and 2018 ce (de la Vega et al. 2021). In our reanalysis-driven simulation, the baseline (1970–1990 ce) difference between these regions was ~ 1‰ and increased to ~ 2‰ in the final decade (2009–2019 ce), thus consistent with the overall increase in the spatial gradient of roughly 1‰.

### Drivers of historical isotopic trends

Hereafter, we focus on δ^15^N_POM_ as the isotopic signature of POM is most closely associated with the isoscape integrated into Arctic food webs. Furthermore, POM is not only consumed by epipelagic species, by also by benthic species as this material sinks through the water column. Trends in δ^15^N_POM_ are therefore important for both epipelagic and benthic food webs.

**Fig. 3:**
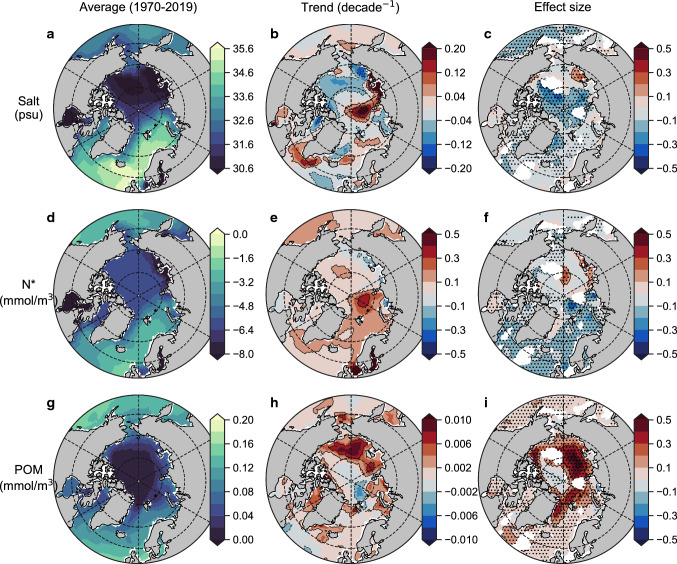
Major environmental drivers of the Arctic Ocean isoscape from the reanalysis-driven simulations. Average values of salinity, N* and particulate organic matter (units nitrogen) over the upper 100 m of the Arctic Ocean over simulation years 1970–2019 ce (**a**, **d**, **g**), their linear multi-decadal trends (**b**, **e**, **h**) and their normalised effect size (unitless) on inter-annual trends in δ^15^N_POM_ (**c**, **f**, **g**). Masked regions in right-hand panels (**c**, **f**, **i**) are those where regression analysis could not be performed with all three variables due to interactive effects between variables (variance inflation factor > 3.0). Stippling in right-hand panels (**c**, **f**, **i**) indicates a significant effect of the variable on δ^15^N_POM_, where the 95% confidence intervals of the effect size do not intersect zero

By applying a multiple linear regression analysis to time series of δ^15^N_POM_ at each model grid cell (see “Methods”; Table 1) we found that the concentration of POM and N* had the strongest effects on isotopic trends (Fig. 3). Although salinity had strong effects in the central Arctic, the very low concentrations of POM and year-round presence of sea ice would result in this region having very little influence on the isotopic signatures integrated within Arctic food webs. Overall, salinity was, therefore, a relatively poor predictor of Arctic δ^15^N_POM_ trends despite some strong regional trends (Fig. 3a–c), while POM and N* had much larger effects and larger footprints. Moreover, their geographic distributions appeared to align with divergent trends between the Pacific- and Atlantic-sector seas.

In the Atlantic sector, N* showed significant, negative effects (Fig. 3d–f), such that an increase in N* was associated with a decrease in δ^15^N_POM_. N* reflects the balance between NO_3_ and PO_4_, with positive values indicating a relative surplus of NO_3_ and negative values representing a relative deficit (Gruber and Sarmiento 1997). N* therefore highlights contrasting water masses of Atlantic and Pacific origin (Fig. 3d), similar to salinity (Tuerena et al. 2021), but unlike salinity will include the effects of N_r_ input while excluding freshwater fluxes. It is therefore notable that both salinity and N* showed negative effects, which indicates that an encroachment of Atlantic water into the region was important for lowering δ^15^N_POM_ values. However, the stronger influence of N* suggested that both increasing Atlantic inflow and increasing N_r_ inputs caused δ^15^N_POM_ declines in the Atlantic sector. In the Pacific-influenced high Arctic, increases in POM were well correlated with increases in δ^15^N_POM_ and showed significant, positive effects (Fig. 3g–i). This relationship is simple; increasing POM primarily corresponds to increasing primary production that enriches NO_3_ and phytoplankton in ^15^N (Karsh et al. 2012; Sigman and Fripiat 2019), and secondarily stimulates sedimentary denitrification that further enriches NO_3_ in ^15^N (Granger et al. 2011).

We performed two additional simulations to definitively quantify the importance of external fluxes of nitrogen to the Arctic Ocean. This included anthropogenic N_r_ deposition and inter-annual changes in riverine freshwater and nutrient fluxes. The importance of anthropogenic N_r_ deposition to the multi-decadal isotopic decline in the Atlantic sector was confirmed. Without the anthropogenic increase in N_r_ deposition, the decline in δ^15^N_POM_ was reduced in magnitude or even reversed (Figs. S8, S9a–c). In the Labrador and Barents Seas, the decline was reduced to 38% and 14% of their magnitude, while in the East Greenland Sea the decline was reversed. Rivers had a minor effect in the Atlantic sector as expected, except to slightly increase δ^15^N_POM_ values. As rivers release nitrogen with a low δ^15^N signature, the only means for rivers to increase δ^15^N_POM_ is to stimulate primary production (Terhaar et al. 2021). In contrast, anthropogenic N_r_ deposition played a minor dampening role in the high Arctic and Pacific-influenced seas (Fig. S9d–f). The contribution of riverine fluxes also had little effect, with the exception of the Southern Beaufort Sea, where the multi-decadal increase in δ^15^N_POM_ appeared to depend on the riverine fluxes that stimulated local primary production. Importantly, the lack of a consistent, overarching role for N_r_ deposition and rivers for the high Arctic (i.e. Pacific-influenced) isoscape indirectly implicated primary production as the major cause of the δ^15^N_POM_ trends there.

### Future changes in the Arctic isoscape

**Fig. 4:**
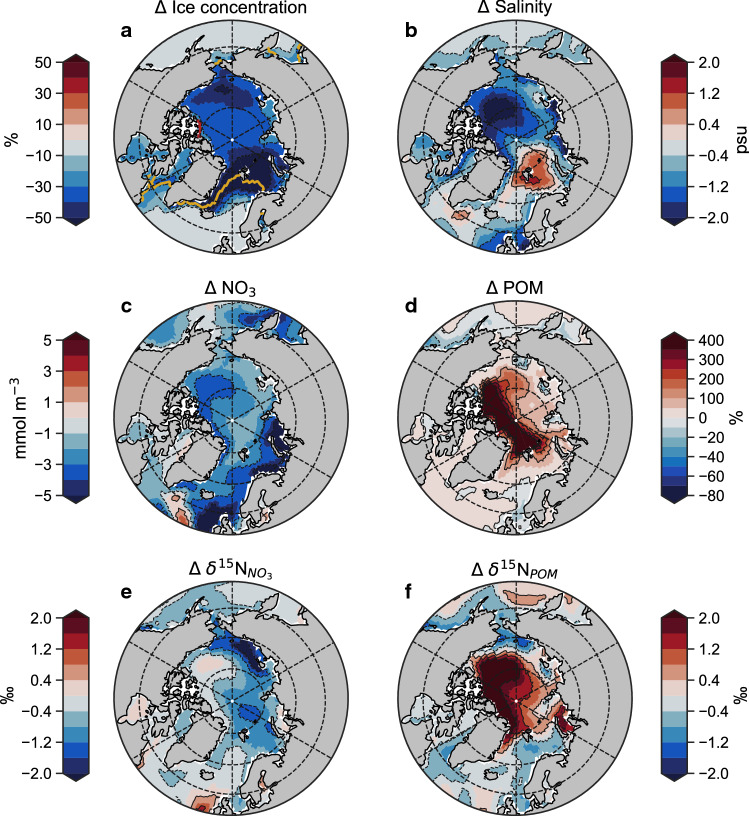
Future changes in surface properties of the Arctic Ocean nitrogen isoscape. Changes (*Δ*) between mean conditions over upper 100 m between 2081–2100 and 1986–2005. **a** Sea ice concentration. **b** Salinity. **c** Concentration of nitrate (NO_3_). **d** Percent change in the concentration of particulate organic matter (POM) in units of nitrogen. **e** Values of δ^15^N (isoscape) of NO_3_. **f** Values of δ^15^N (isoscape) of POM in units of nitrogen. All values come from the emissions-driven simulation from preindustrial to future conditions (1801–2100)

The loss of sea ice, increasing Atlantic inflow, increase in primary production and drawdown of nitrate that were observed in our reanalysis-driven simulation were also observed in emissions-driven simulations of the future Arctic (Fig. 4). By the end of the twenty first century (2081–2100 ce), annual mean sea ice had declined by over 50% of its historical concentration (1986–2005 ce) and summer sea ice was almost non-existent (red contour in Fig. 4a). A signature of increasing Atlantic presence in the Irminger, Greenland and Barents Seas was evident by increases in salinity of around 1 psu. Nitrate concentrations were reduced markedly throughout the Arctic and POM increased by over 400% (fivefold) in the high Arctic, which include the northern Barents Sea, Fram Strait, Canadian Archipelago and the Beaufort Gyre.

Widespread declines in $\updelta^{15} {\text{N}}_{{{\text{NO}}_{3} }}$ of between 0.4‰ and 1.2‰ developed by 2081–2100, while δ^15^N_POM_ increased in the high Arctic by up to 2‰ (Fig. 4e, f). In theory, opposing trends in $\updelta^{15} {\text{N}}_{{{\text{NO}}_{3} }}$ and δ^15^N_POM_ in the high Arctic can be explained by the response of phytoplankton to very low concentrations of NO_3_. Once NO_3_ is at very low levels, the preference for ^14^N over ^15^N during assimilation is reduced (Karsh et al. 2012). Overall, this means that the $\updelta^{15} {\text{N}}_{{{\text{NO}}_{3} }}$ signal declines as organic matter with a heavier δ^15^N signature is produced under very low nitrogen availability in the future Arctic.

**Fig. 5:**
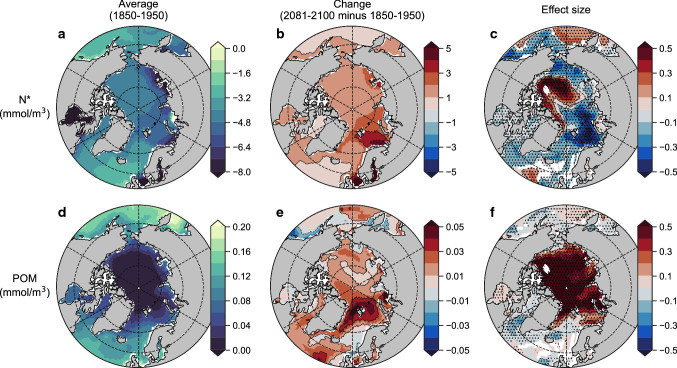
Major environmental drivers of the Arctic Ocean isoscape from the emissions-driven simulations. Average values of N* and particulate organic matter (units nitrogen) over the upper 100 m of the Arctic Ocean over simulation years 1850–1950 ce (**a**, **d**), their linear multi-decadal trends (**b**, **e**), and their normalised effect size (unitless) on inter-annual trends in δ^15^N_POM_ (**c**, **f**). Masked regions in right-hand panels (**c**, **f**) are those where regression analysis could not be performed with all three variables due to interactive effects between variables (variance inflation factor > 3.0). Stippling in right-hand panels (**c**, **f**) indicates a significant effect of the variable on δ^15^N_POM_, where the 95% confidence intervals of the effect size do not intersect zero

**Fig. 6:**
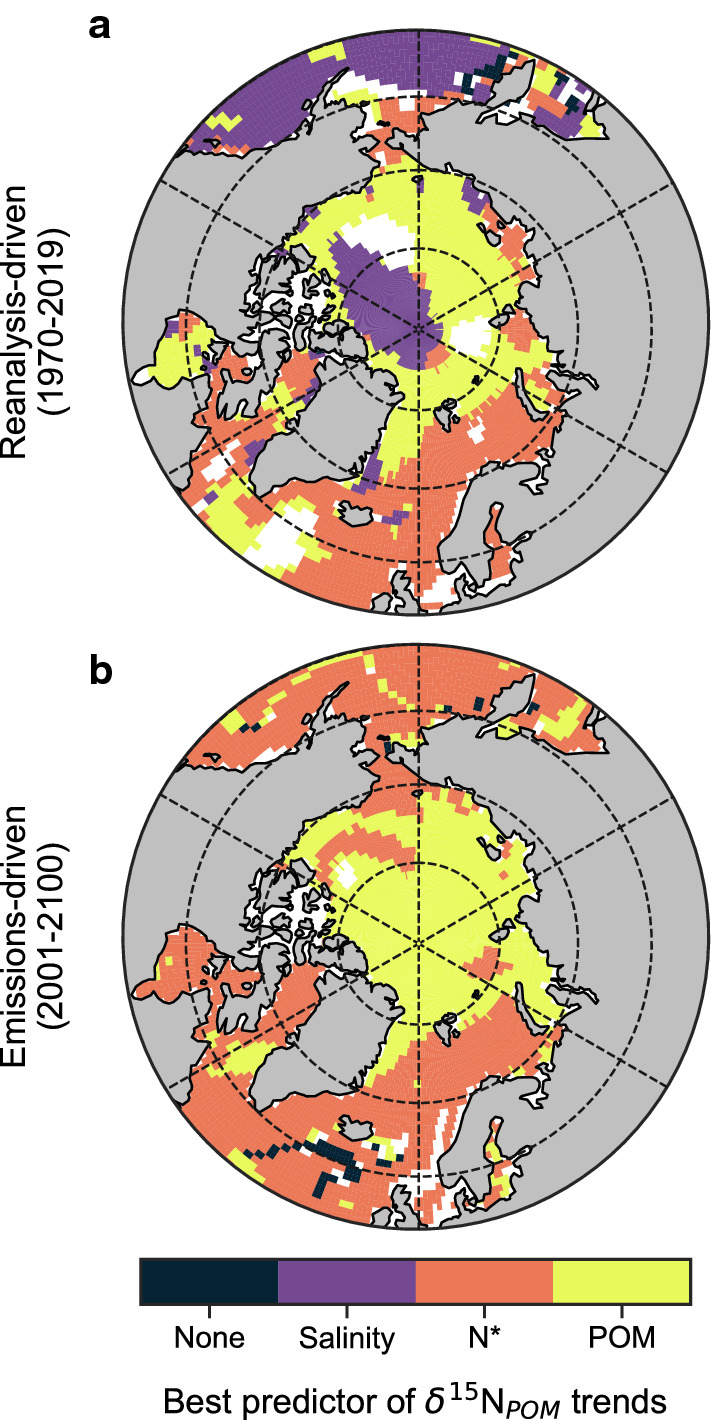
Best predictor of temporal trends in the Arctic isoscape. Results of a multiple linear regression analysis using predictors of salinity (psu), N* (mmol m^−3^), and POM (mmol m^−3^) and the response of δ^15^N_POM_. All variables were averaged over the upper 100 m and over the year, such that trends are inter-annual. N* = NO_3_ − PO_4_*16. POM is particulate organic matter in terms of nitrogen. White spaces are regions where the predictor variables showed significant interactive effects (variance inflation factor > 3.0) and where multiple linear regression was not performed. Salinity was removed as a predictor in the emissions-driven scenario due to variance inflation factors between it and N*

A multiple linear regression analysis on these emissions-driven simulations using only N* and POM as predictor variables (salinity was excluded due to strong interactive effects with N*; Table 1) supported this logic as POM was the strongest influence on the high Arctic isoscape, while N* was clearly most important in the Atlantic-sector seas (Fig. 5). Decreases in both $\updelta^{15} {\text{N}}_{{{\text{NO}}_{3} }}$ and δ^15^N_POM_ in the Atlantic-sector seas were coincident with increases in N*, which was of greatest influence on the isoscape outside of the high Arctic (Fig. 5a–c). The Atlantic-sector seas were thus affected by both increasing Atlantic water presence, evident by salinity increases (Fig. 4b), and coincident increases in N_r_ delivered both directly and indirectly via lateral transport. Meanwhile in the high Arctic where δ^15^N_POM_ increased, POM was of strong positive influence and dominated trends (Fig. 5d–f). An increasing isotopic gradient between the high Arctic and the Atlantic-sector seas was therefore driven by similar mechanisms in both reanalysis- and emissions-driven simulations (Fig. 6).

To determine the cause for the decline of δ^15^N_POM_ in sub-Arctic seas, we performed an additional set of idealised simulations (Fig. S10) where the increase in anthropogenic N_r_ deposition was removed or fractionation during phytoplankton assimilation of inorganic nitrogen was removed (set at 0‰). We focussed on average trends within the East Greenland Sea and the Beaufort Gyre (Fig. S8) to contrast a sub-Arctic sea affected by Atlantic inflow with a high Arctic region strongly affected by primary production. In the East Greenland Sea, parallel declines in $\updelta^{15} {\text{N}}_{{{\text{NO}}_{3} }}$ and δ^15^N_POM_ were driven by increasing N_r_ deposition, but were also affected by increasing Atlantic inflow that brought low $\updelta^{15} {\text{N}}_{{{\text{NO}}_{3} }}$ signals northwards. However, this effect was absent when anthropogenic N_r_ deposition was not included, which showed little difference with the preindustrial control as the increase in primary production that raised δ^15^N was compensated for by the greater Atlantic inflow that reduced δ^15^N. Meanwhile, the key role of phytoplankton primary production for driving an increase in δ^15^N_POM_ in the Beaufort Gyre was confirmed. Finally, the transition to nitrogen limitation in the Beaufort Gyre was fingerprinted by the $\updelta^{15} {\text{N}}_{{{\text{NO}}_{3} }}$ trend where an initial increase is followed by a decrease after 2050 ce as the strength of phytoplankton fractionation declined as NO_3_ levels became more depleted.

## Implications

This study highlights strong spatiotemporal variations in the Arctic δ^15^N baseline. These results are in line with a meta-analysis of Arctic stable isotope studies (Hoondert et al. 2021) and a compilation of Arctic $\updelta^{15} {\text{N}}_{{{\text{NO}}_{3} }}$ measurements (de la Vega et al. 2021) that show strong inter-regional differences, with higher δ^15^N values in Pacific-influenced waters and lower values in the more Atlantic-influenced waters. Our model reconstructs these spatial gradients, but further suggests that these inter-regional differences are amplifying now and into the future. The amplification is driven by increasing phytoplankton production (Lewis et al. 2020), Pacific and Atlantic inflows (Spielhagen et al. 2011; Woodgate 2018; Oziel et al. 2020) and anthropogenic nitrogen inputs into the system (Hauglustaine et al. 2014; Yang and Gruber 2016), which together elevate the Pacific sector baseline and depress the Atlantic sector baseline. It must be stressed, however, that our modelled δ^15^N isoscape is an imperfect approximation of reality. A comparison between modelled and measured $\updelta^{15} {\text{N}}_{{{\text{NO}}_{3} }}$ at each year from 1970 to 2018 ce revealed that our model consistently underestimated observed values by between 0.5 and 2‰ (average of 1.25‰). Furthermore, the coarse resolution of our ocean model, which could not resolve eddy-driven transport and mixing in this dynamic region (Nurser and Bacon 2014), almost certainly meant that the model severely underestimated temporal and spatial variability. In effect, our simulated isoscape is prone to significant error over fine spatial (< 200 km) and temporal (< 3 months) scales and outputs from this study should be used with caution at these scales. However, over broader spatiotemporal scales encompassing seasons, years and oceanographic provinces, the simulated isoscape gradients presented in this study are representative.

The shifting δ^15^N baseline in the Arctic has major implications for the study of Arctic food webs. δ^15^N of bulk tissue is classically used to estimate trophic position (Post 2002), but is highly influenced by change at the δ^15^N baseline. It is therefore difficult to determine if a change in bulk δ^15^N values over time or space represents a change in that species trophic position without a good understanding of how the baseline also varies in time and space. Spatial and temporal variations in the nitrogen isoscape must, therefore, be considered to accurately interpret spatial and/or temporal changes in trophic position of consumers when using δ^15^N of bulk tissue (de la Vega et al. 2021). Trophic position is a fundamental property of ecological communities, reflecting integrated changes in ecosystems. Trophic position of top predators is an expression of food chain length (Post 2002) and can be used as an indicator of food web complexity (Post and Takimoto 2007), efficiency of energy transfer through the food web (Lindeman 1942), fisheries dynamics (Bourdaud et al. 2016) and contaminant bio-accumulation (Jæger et al. 2009; Braune et al. 2015). Accurate estimation of trophic position of predators is, therefore, crucial to manage and protect ecosystems, especially in the rapidly changing Arctic.

Fortunately, the use of compound specific stable nitrogen isotopes has recently developed and can overcome the challenge of a shifting baseline by targeting specific amino acids that show minimal fractionation during trophic transfer (McMahon and Newsome 2019), therefore tracking the δ^15^N baseline within predator tissues (de la Vega et al. 2021). Compound specific isotope analyses, therefore, have great potential for studies of migration patterns and movements of predators, as demonstrated using stable carbon isotopes (Hobson 1999; MacKenzie et al. 2011; Bird et al. 2018). Thus, while the divergent trends between Pacific and Atlantic sectors may complicate longitudinal studies of food web structure, they may aid studies of migration and foraging patterns that rely on strong spatial gradients to pinpoint area use (McMahon and Newsome 2019). However, even these must account for spatiotemporal changes to ensure that area use is accurately allocated. Ultimately, the changing nitrogen isoscape must be constrained to accurately trace changing ecological interactions in the face of borealisation and other climate-driven shifts within Arctic ecosystems.

The consistent message from both the reanalysis- (historical) and emissions-driven (future) modelling experiments is that the ongoing changes to the Arctic isoscape will continue and expand in the future due to their common anthropogenic driver. Rapid warming and sea ice loss (Meredith et al. 2019) increased δ^15^N_POM_ in the Pacific-influenced high Arctic, while increasing Atlantic inflow (Spielhagen et al. 2011; Oziel et al. 2020) and anthropogenic inputs (Galloway 2014; Hauglustaine et al. 2014; Yang and Gruber 2016) decreased δ^15^N_POM_ in the Atlantic sector, over-riding the tendency for increasing primary production (Lewis et al. 2020) to increase δ^15^N_POM_ here. Hence, both sectors of the Arctic experienced increases in primary production in our simulations, as is the case in other models (Bindoff et al. 2019), but experienced divergent trends in the isoscape due to additional drivers in the Atlantic-sector seas. While we have not yet accounted for certain drivers, such as inter-annual changes in terrestrial nutrient fluxes linked to rivers and continental erosion (Terhaar et al. 2021) and the role played by an increase in Pacific inflow (Woodgate 2018), our results provide a clear indication that anthropogenic impacts are integrated into the nitrogen isoscape and underpinned by a distinct and divergent spatial response. In fact, any increase in Pacific inflow would exacerbate the spatial divergence because the Pacific δ^15^N endmember is high (Somes et al. 2010; Buchanan et al. 2019), which lends additional confidence in the rigour of our simulated trends. Our work highlights how environmental changes in the Arctic Ocean impact the nitrogen isoscape. As these changes are transferred along the food chain to higher predators, they must be accounted for when using stable isotopes to study food webs and will be essential for monitoring the consequences of phytoplankton productivity and community composition changes in the coming years.

## Supplementary Information

Below is the link to the electronic supplementary material.

Supplementary file1 (PDF 724 kb)

## Data Availability

Simulated isoscapes and the predictors used in the multiple linear regression analysis in both reanalysis-driven and emissions-driven simulations are freely available at https://doi.org/10.5281/zenodo.5359263.
