# Supplementary material for: Oceanographic and biogeochemical drivers cause divergent trends in the nitrogen isoscape in a changing Arctic Ocean
Source: Ambio. 2021 Oct 9;51(2):383–97. doi: 10.1007/s13280-021-01635-6 (PMC8692545; doi:10.1007/s13280-021-01635-6)
Supplement: Supplementary file 1 — Supplementary file1 (PDF 724 kb) [file 13280_2021_1635_MOESM1_ESM.pdf]

Ambio

Supplementary Information
This supplementary information has not been peer reviewed.

Title: **Oceanographic and biogeochemical drivers cause divergent trends in the nitrogen isoscape in a changing Arctic Ocean**

Appendix S1 – isotopic routines

The nitrogen isotopic routines carry ^{15}N through nitrate (NO_3), ammonium (NH_4), both phytoplankton types, both zooplankton types, small and large particulate organic matter, and dissolved organic matter.

The isotopic signature of nitrogen is expressed in delta notation in units of per mil ‰, where:

$$\delta^{15}N = \left(\frac{\left(\frac{^{15}\mathrm{N}}{^{14}\mathrm{N}}\right)^{sample}}{\left(\frac{^{15}\mathrm{N}}{^{14}\mathrm{N}}\right)^{standard}} - 1 \right) \cdot 1000 \qquad (1)$$

$$\left(\frac{^{15}\mathrm{N}}{^{14}\mathrm{N}}\right)^{standard} = 0.003676 \qquad (2)$$

Key fractionation processes take place during NO_3 and NH_4 assimilation by phytoplankton, denitrification in both the sediments and water column, ingestion of prey by zooplankton, and excretion of NH_4 by zooplankton.

Key sources of ^{15}N are atmospheric reactive nitrogen (N_r) deposition, riverine dissolved inorganic nitrogen, and organic nitrogen fixed by diazotrophs (nitrogen fixers).

All fractionation processes of nitrogen isotopes are biologically mediated and all follow the same formula. Here, we illustrate the formula of nitrogen isotope fractionation using phytoplankton assimilation of NO_3 into biomass during primary production.

$$^{15}N_{t+1}^{phy} = {}^{15}N_t^{phy} + NO_3^{assimilated} \cdot \left(\frac{^{15}\mathrm{N}}{^{14}\mathrm{N}}\right)^{NO_3} \cdot \alpha_{phy} \qquad (3)$$

$$^{15}N_{t+1}^{NO_3} = {}^{15}N_t^{NO_3} - NO_3^{assimilated} \cdot \left(\frac{^{15}\mathrm{N}}{^{14}\mathrm{N}}\right)^{NO_3} \cdot \alpha_{phy} \qquad (4)$$

For each reaction in the nitrogen cycle involving fractionation, the fractionation factor (α) has been experimentally determined by measuring the isotopic ratios of the products and reactants:

$$\alpha = \left(\frac{\left(\frac{^{15}\mathrm{N}}{^{14}\mathrm{N}}\right)^{product}}{\left(\frac{^{15}\mathrm{N}}{^{14}\mathrm{N}}\right)^{reactant}} \right) \qquad (5)$$

Isotope fractionation factors are close to one and so are often represented in per mil units using the ε notation, where:

$$\varepsilon = (1 - \alpha) * 1000 \qquad (6)$$

The fraction factors for most reactions are assumed constant, with the exception of NO_3 and NH_4 assimilation by phytoplankton. Their default values are:

- Phytoplankton assimilation (α_{phy}) = 0.995 (ε_{phy} = 5 ‰)
- Water column denitrification (α_{wc}) = 0.975 (ε_{wc} = 25 ‰)
- Sedimentary denitrification (α_{sed}) = 0.997 (ε_{sed} = 3.0 ‰)
- Nitrification (α_{nit}) = 1.0 (ε_{nit} = 0.0 ‰)
- Zooplankton ingestion (α_{ing}) = 1.001 (ε_{ing} = -1.0 ‰)
- Zooplankton excretion (α_{exc}) = 0.994 (ε_{exc} = 6.0 ‰)

Here, positive ε values indicate an enrichment in ^{15}N within the reactant and a depletion in the product. These values are the result of laboratory and field-based investigations of nitrogen isotope dynamics and are summarised by Sigman & Fripiat (2019).

Fractionation during the assimilation of NO_3 and NH_4 by phytoplankton vary according to the ratio of demand and supply of each nutrient. If demand is high but supply is low, such that the amount of nitrogen available to phytoplankton is limiting, then α_{phy} approaches one ($\epsilon_{phy} \rightarrow 0$ ‰). If, however, NO_3 or NH_4 are in high concentrations and/or demand is low, then fractionation proceeds at its maximum value, set at $\alpha_{phy} = 0.995$ (($\epsilon_{phy} = 5.0$ ‰). This utilisation effect is calculated by dividing the NO_3 (NH_4) required by the NO_3 (NH_4) available, and is then multiplied against 5.0 ‰.

Sources of ^{15}N are atmospheric N_r deposition, riverine dissolved inorganic nitrogen, and organic nitrogen fixed by diazotrophs (nitrogen fixers). The signatures of these sources are prescribed using input files during simulations. The default $\delta^{15}N$ signatures of these sources are as follows:

- Atmospheric N_r deposition $\delta^{15}N_{NO3}$ = -4.0 ‰
- Riverine $\delta^{15}N_{NO3}$ = 2.0 ‰
- Diazotrophy $\delta^{15}N_{org}$ = -1.0 ‰

Appendix S2 – model assessment

Isotopic signature of NO_3 ($\delta^{15}N_{NO3}$)

We used a global compilation of $\delta^{13}N$ of nitrate ($\delta^{15}N_{NO3}$) measurements presented in Rafter et al. (2019), supplemented with data from the Arctic Ocean, to compare with the model. This measurement dataset contained 13,096 measurements from all ocean basins over years 1971 to 2018 Common Era (CE). We made one-to-one comparisons with simulated $\delta^{15}N_{NO3}$ from the historical simulations by gridding the observations onto the model grid at the appropriate year and month of sampling. If multiple measurements occurred in the same bin, these were averaged. Due to averaging and masking at model land tiles, the model-data comparison involved a total of 8,792 comparisons covering approximately 0.8 % of the ocean model volume (Fig. S1).

Simulated $\delta^{15}N_{NO3}$ provided an adequate overall fit to the *in situ* $\delta^{15}N_{NO3}$ measurements (Fig. S1-S2). A global correlation of 0.62 and regional correlations ranging from -0.38 to 0.84 revealed the difficulty in reproducing *in situ* $\delta^{15}N_{NO3}$ measurements. The worst regional fit, with a correlation of -0.34, was for the Indian Ocean, where the model oxygen field misrepresented the oxygen minimum zones in the Arabian Sea and Bay of Bengal. Because most observations of $\delta^{15}N_{NO3}$ are from the Arabian Sea, the misplacement of the low oxygen zone had a strong effect on the regional fit. In fact, measurements programs are biased towards sampling within or near to the oxygen minimum zones where denitrification is active, but where the strongest gradients in $\delta^{15}N_{NO3}$ exist in the modern ocean. The best regional fit therefore, with a correlation of 0.84, was for the Southern Ocean where the $\delta^{15}N_{NO3}$ distribution is primarily affected by the physical positioning of water masses and summertime productivity, and the spatial gradients are weaker. All regions suffered from subdued variance compared with the data, which was evident by low normalised standard deviations, but is to be expected when comparing measurements, which are prone to high frequency variability, to monthly-averaged model output.

Next, we assessed the model-data correlation and model bias for each major region and for each year of the simulation (Fig. S3). We accounted for seasonality by ensuring that comparisons were occurring at the correct month. This analysis revealed strong correlations in the Southern Ocean, the absence of a correlation in the Indian Ocean, and more variable correlations in the Atlantic and Pacific. The Arctic Ocean contained one year of poor model-data fit (2014 CE), but returned correlations exceeding 0.6 for other years. Global correlations tended to exceed 0.5, with those lower than 0.5 dragged downwards due to poor agreement in the Indian ocean. All basins contained simulated $\delta^{15}N_{NO3}$ values that underestimated the measured values, typically by between 0.5 and 1.5 ‰. Importantly, the underestimation appeared consistent across basins, ensuring that the inter-basin gradients of $\delta^{15}N_{NO3}$ were well reproduced.

Finally, we visually assessed the simulated $\delta^{15}N_{NO3}$ of the upper 100 metres in the Arctic and compared with a compilation of data from oceanographic cruises (Fig. S4). The strong regional contrast between the Pacific-influenced and Atlantic-influenced waters is evident, with Pacific seawater being enriched in ^{15}N due to active water column denitrification in this basin. Atlantic isotopic signatures are depleted due to active nitrogen fixation and atmospheric nitrogen deposition in the North Atlantic, which introduces low $\delta^{15}N$ signatures to the nitrate in this basin. Finally, the model-derived values underestimate the measured values in the upper water column of the Arctic, but do so consistently such that the spatial gradients are conserved.

Arctic and subarctic conditions

To determine the performance of the model for simulating surface and circulatory conditions in the Arctic and subarctic North Atlantic, we used observation-based products of sea ice concentration, sea surface temperature (SST) and sea surface height (SSH) to compare with the model output. Observations of sea ice concentration and SST were provided by the National Oceanic and Atmospheric Administration's Optimal Interpolation SST version 2.1 (OISSTv2.1). The OISSTv2.1 blends remotely sensed daily SST and sea ice concentration from 1981 to present day to produce a global product on a $1/4^{th}$ degree grid (Reynolds et al. 2007). For SSH, we used output from the Ocean Reanalysis System 4 (ORA-S4) as part of the European Centre for Medium-Range Weather Forecasts (Balmaseda et al. 2013). The ORA-S4 assimilated remotely-sensed and in situ observations of temperature, salinity and sea level anomaly to predict historical ocean conditions from 1959-present.

These datasets were regridded to a 1° by 1° horizontal grid for direct comparison with the model output. Monthly mean sea ice concentrations and SST in the Arctic domain were compared directly using the nonparametric Spearman's rank correlation, owing to non-normal distributions of these variables. Model-data correlations in sea ice ($r=0.97$) and SST ($r=0.89$) were strong, but a seasonal lag was apparent in which the model warmed and cooled too late each year. Meanwhile, SSH was compared in terms of its inter-annual variability. We computed the Subpolar Gyre Index in the North Atlantic (Koul et al. 2020), the North Atlantic Oscillation, and the first empirical orthogonal function in the Arctic domain. We compared the timeseries of these modes of variability between the ORA-S4 and the model using linear correlation (Pearson's). The model-data comparisons were strong for the Subpolar Gyre Index ($r=0.85$), the North Atlantic Oscillation ($r=0.78$), and the principal component timeseries of the first empirical orthogonal function over the Arctic ($r=0.85$).

Figures

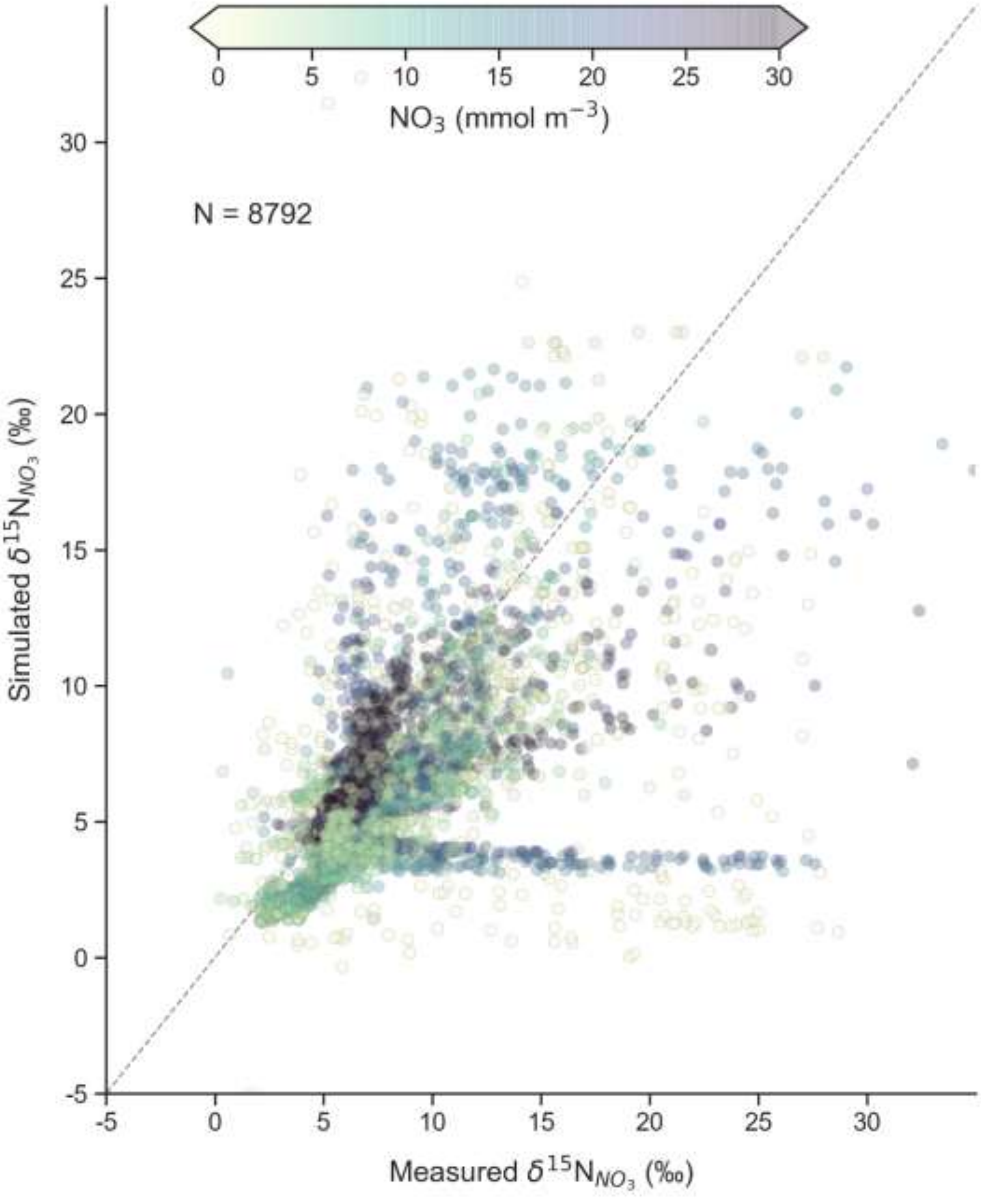

Figure S1. Direct comparison of measured and modelled $\delta^{15}N_{NO3}$, coloured by NO_3 concentration.

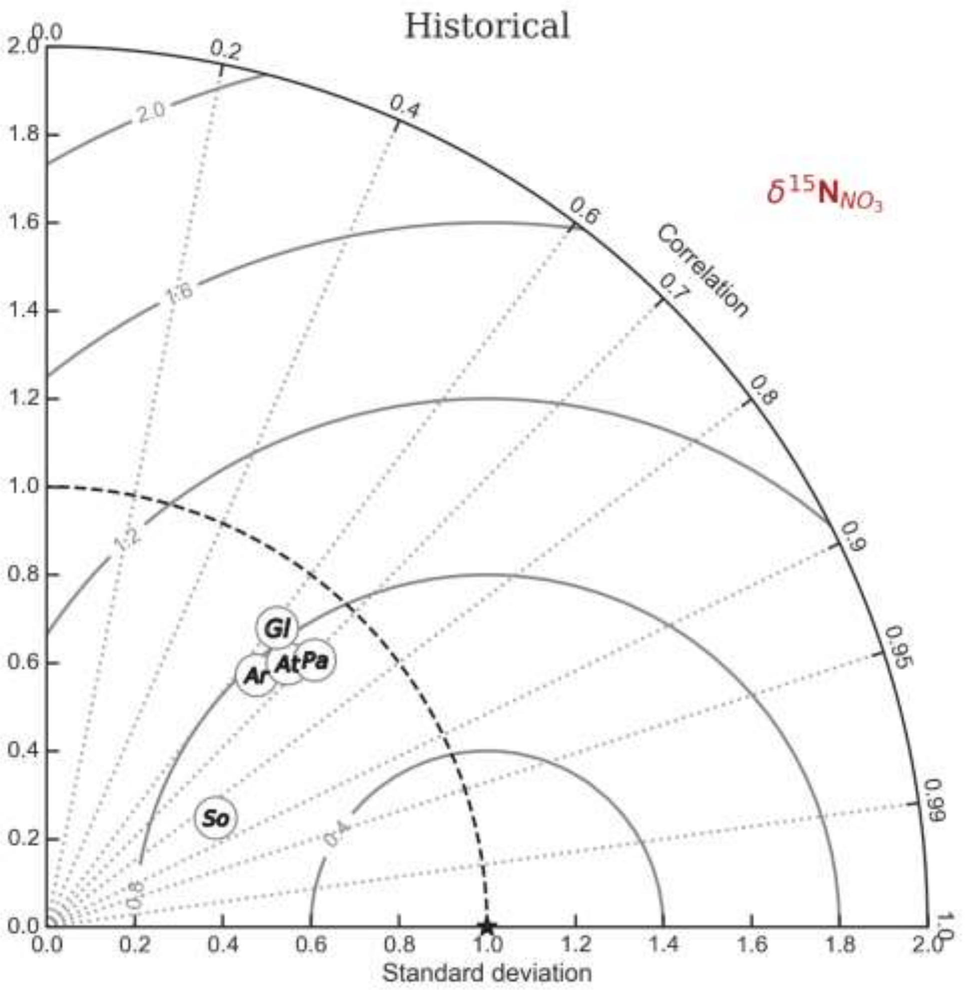

Figure S2. Taylor diagram (Taylor 2001) summarising the model-data fit for $\delta^{15}N_{NO3}$. A perfect match between the model and the data would place a marker on top of the star marker, with a correlation of 1.0, a normalised standard deviation of 1.0, and a root mean square error of 0.0. Correlations (Pearson's r) are represented by radii. Normalised standard deviations are relative to the black dashed line, such that normalised standard deviations less than 1.0 plot below this line. Contours of constant root mean square error are represented by the solid grey lines. Gl = Global; So = Southern Ocean; At = Atlantic; Pa = Pacific; Ar = Arctic. Note that the Indian Ocean does not feature because of a negative correlation.

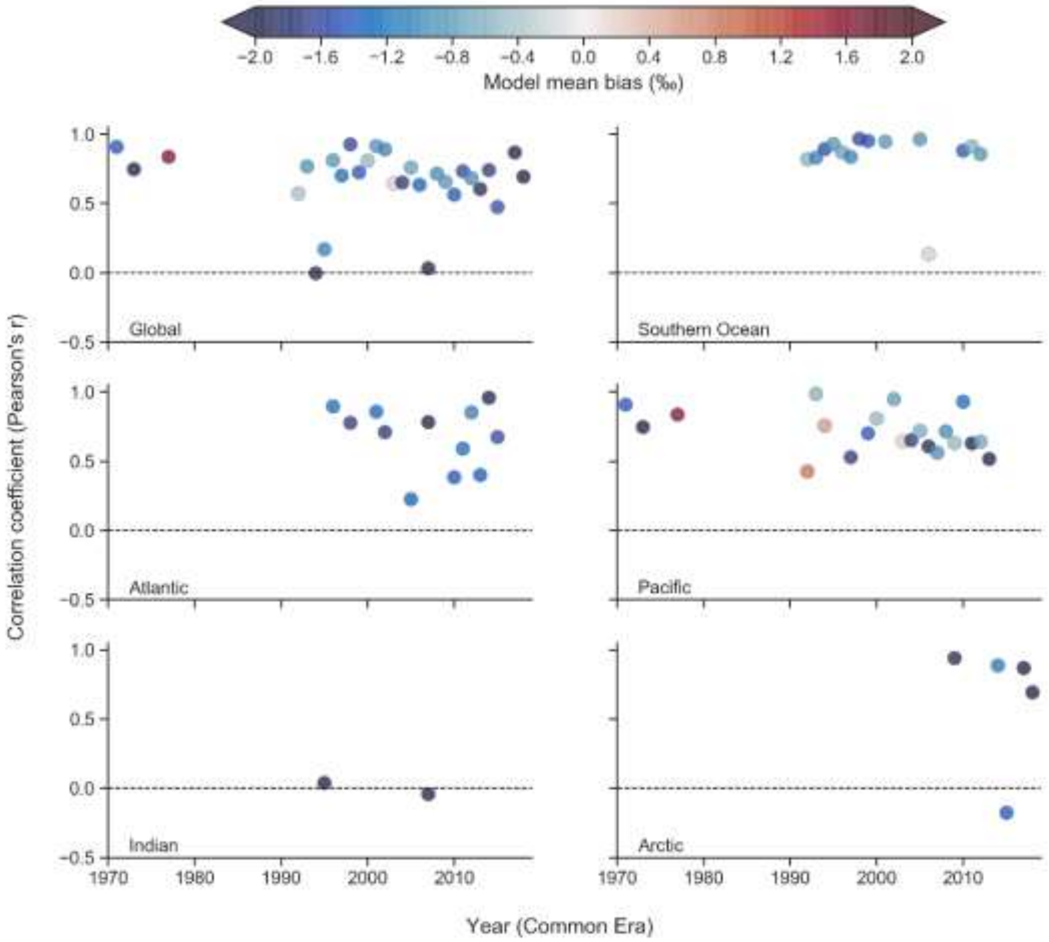

Figure S3. Correlations and model biases with the global compilation of $\delta^{15}N_{NO3}$ data for different regions and different years. Comparisons take seasonality into account by comparing observations and model output at the correct month.

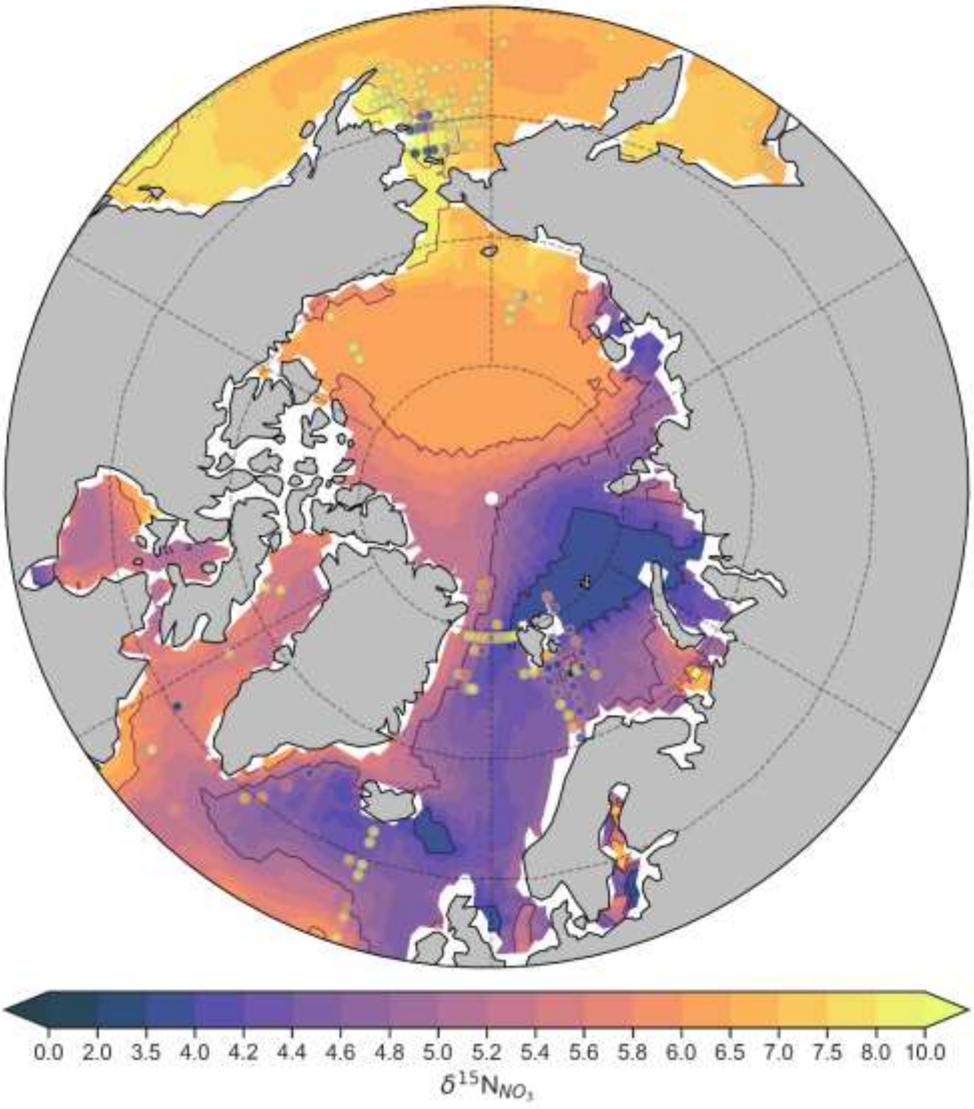

Figure S4. Annual mean $\delta^{15}N_{NO3}$ (shading) and a compilation of measurements (markers) made during Arctic oceanographic cruises.

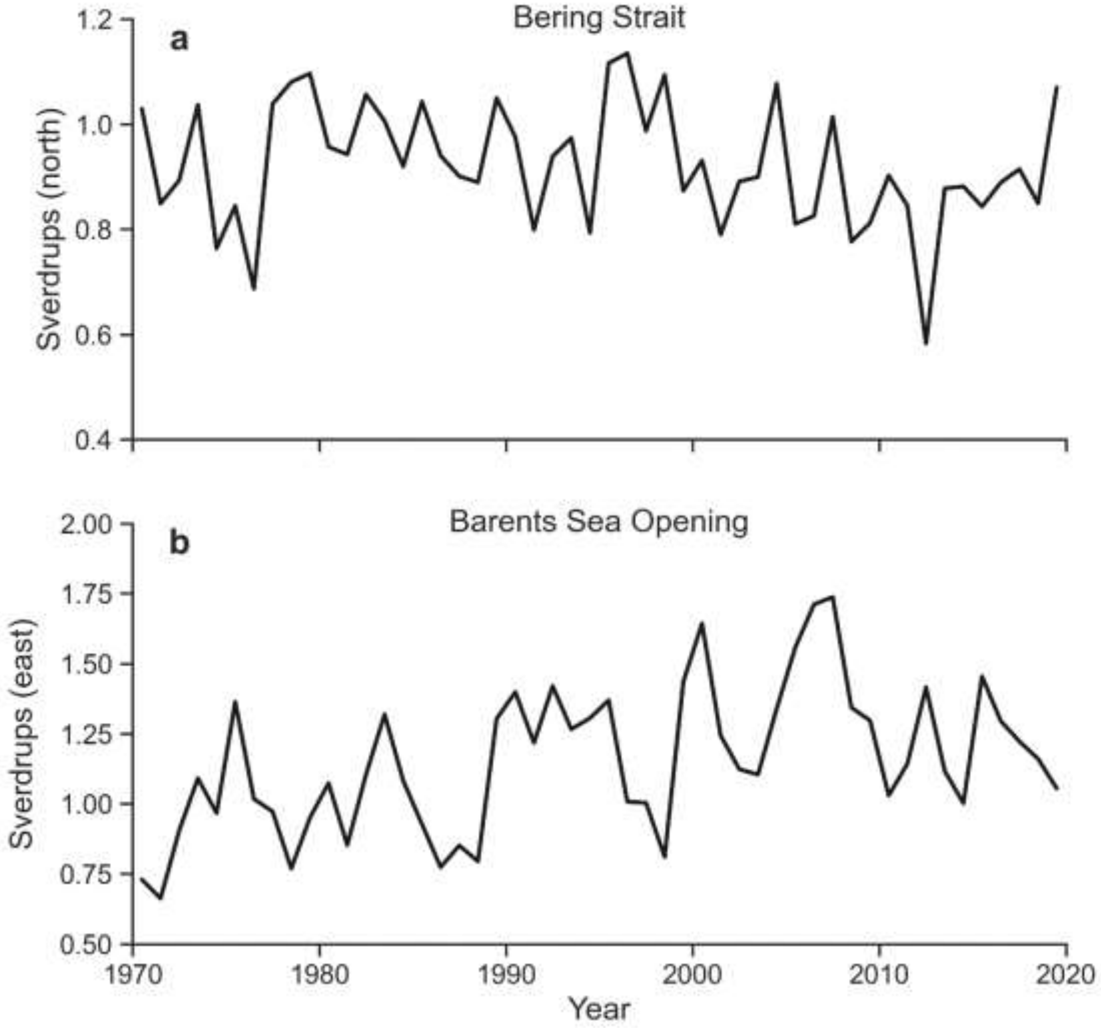

Figure S5. Depth integrated transport of water across the Bering Strait at 65°N (**a**) and the Barents Sea Opening at 20°E (**b**). For the Barents Sea Opening, we select only transports within water masses of Atlantic origin, with potential temperature greater than 2°C and salinity greater than 34.5 psu.

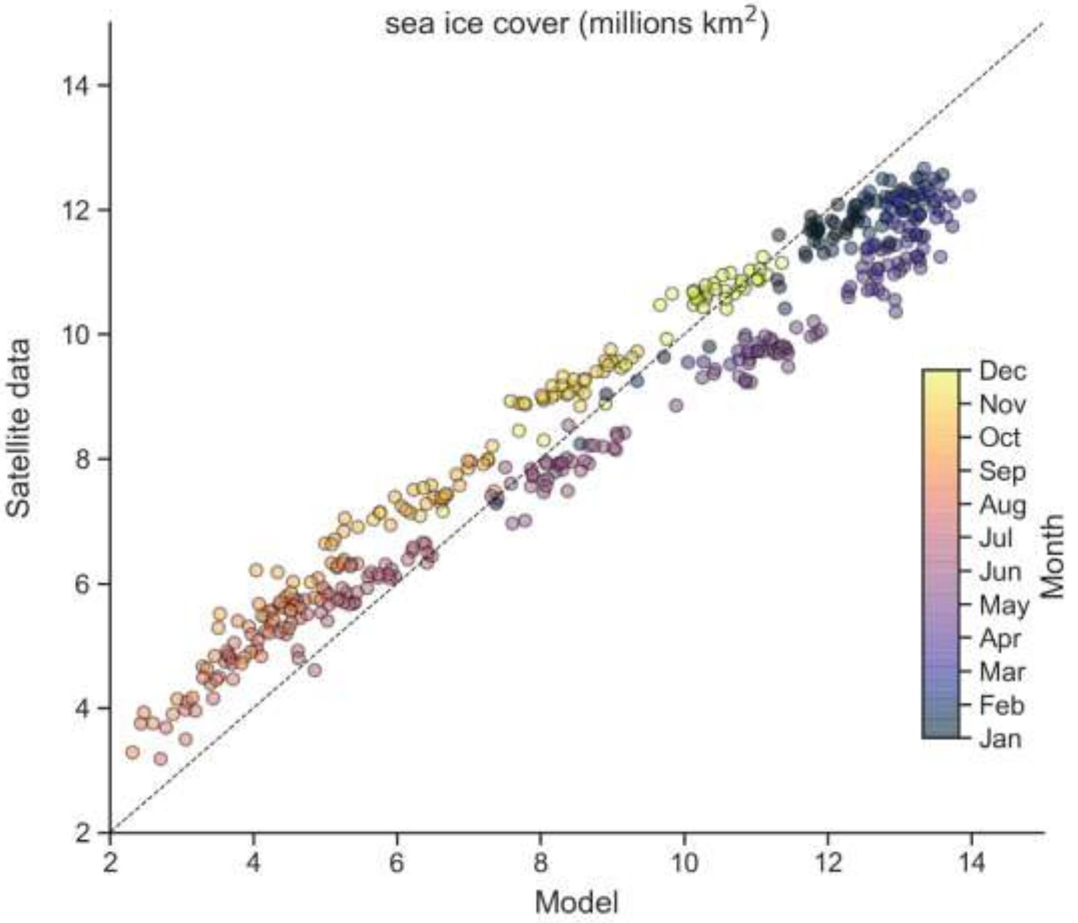

Figure S6. Direct comparison of monthly Arctic sea ice extent between the satellite record and our reanalysis simulation from December 1981 to December 2018. Satellite record comes from the National Oceanographic and Atmospheric Administration (NOAA) Optimal Interpolation version 2.1 (Reynolds et al., 2007).

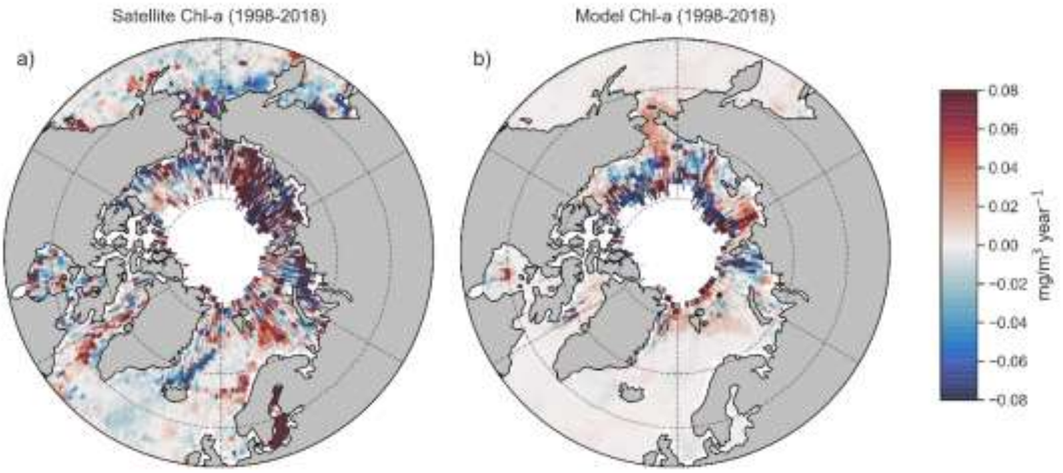

Figure S7. Linear inter-annual trends in remotely sensed (**a**) and simulated (**b**) Arctic Ocean surface chlorophyll-a between 1998 and 2018.

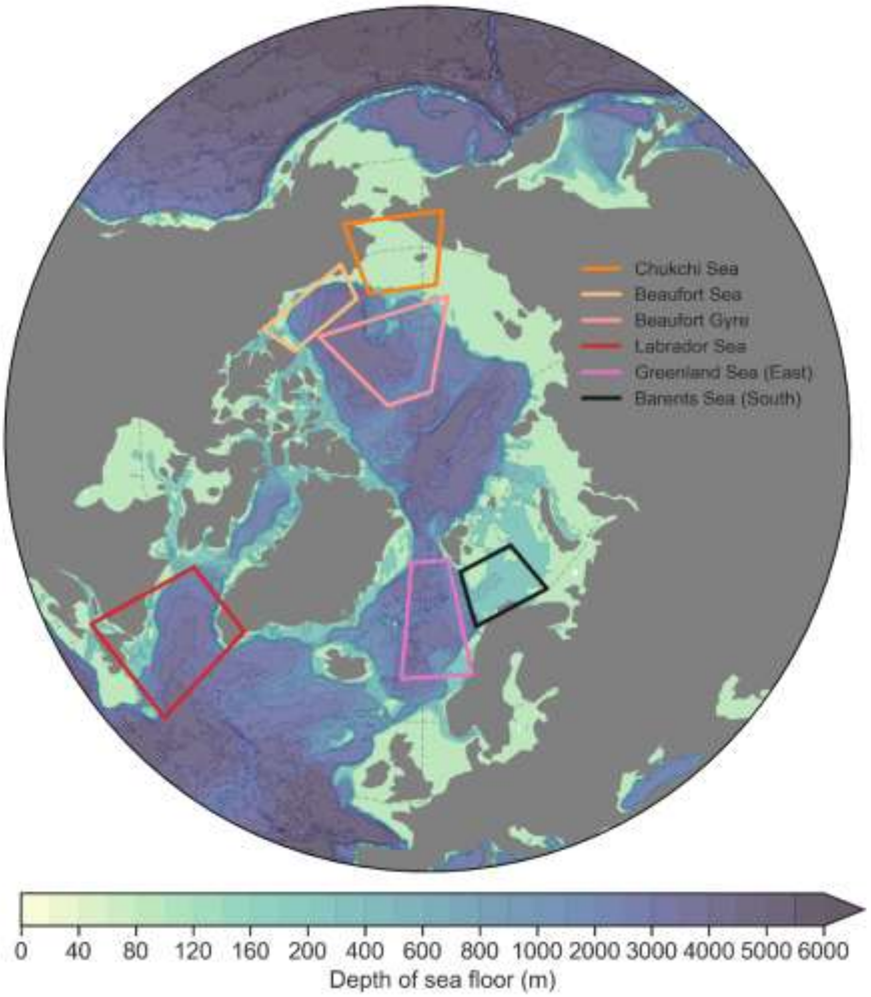

Figure S8. Regions of interest for multiple linear regression analysis. In each region outlined by the different coloured squares, important oceanographic and biogeochemical variables were selected to help explain temporal variations in the isoscape. Bathymetry is shown in by the shading.

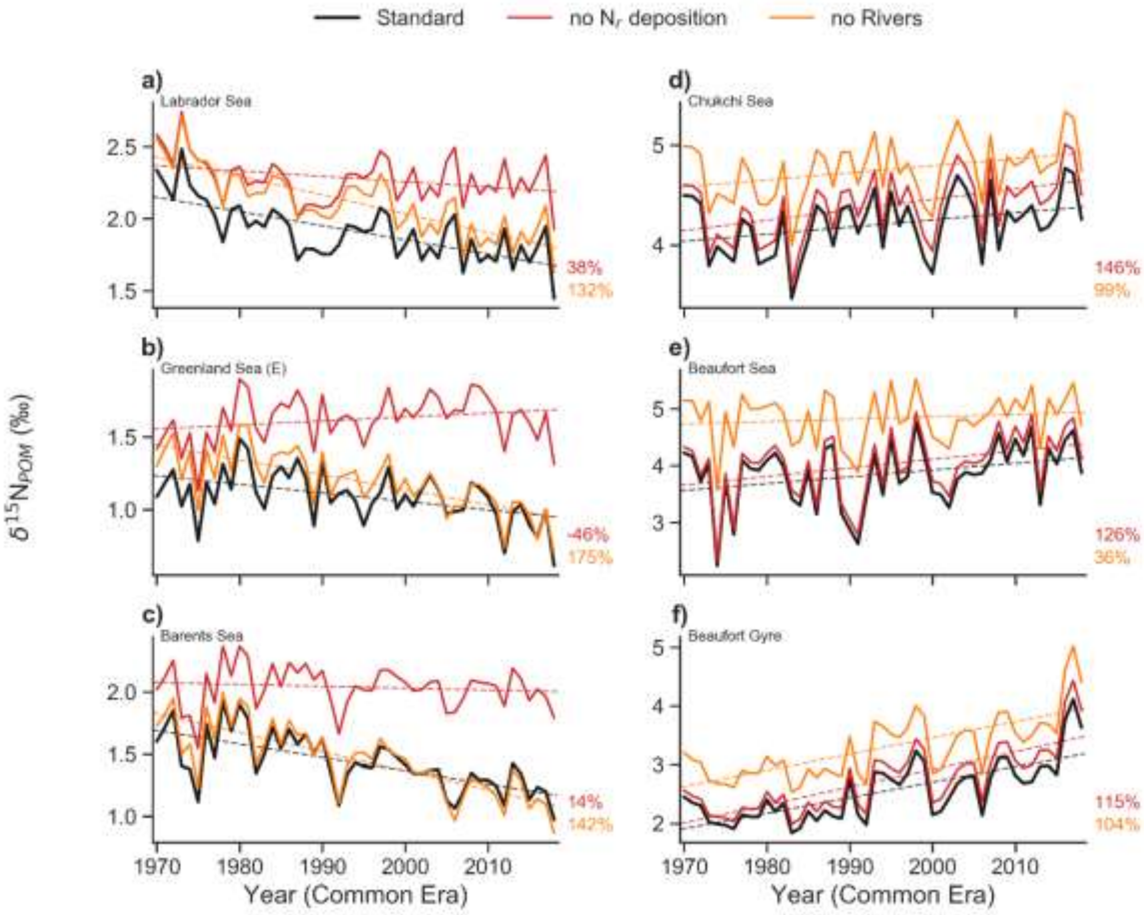

Figure S9. Spatially averaged time series of $\delta^{15}N_{POM}$ in selected regions from the Atlantic (left) and Pacific (right) sectors. Historical trends produced by the full model with both N_r deposition and riverine fluxes is shown in the bold black line. Parallel experiments without each forcing is shown by the red and orange lines. The change in the standard trend caused by the loss of each forcing is shown by the numbers on the right of each panel. For example, the exclusion of anthropogenic N_r deposition reduces the downward, linear trend in $\delta^{15}N_{POM}$ to 38% of its magnitude, while the exclusion of riverine freshwater and nutrient fluxes increases the decline to 132% of its magnitude. Regions are shown in Supplementary Figure 8.

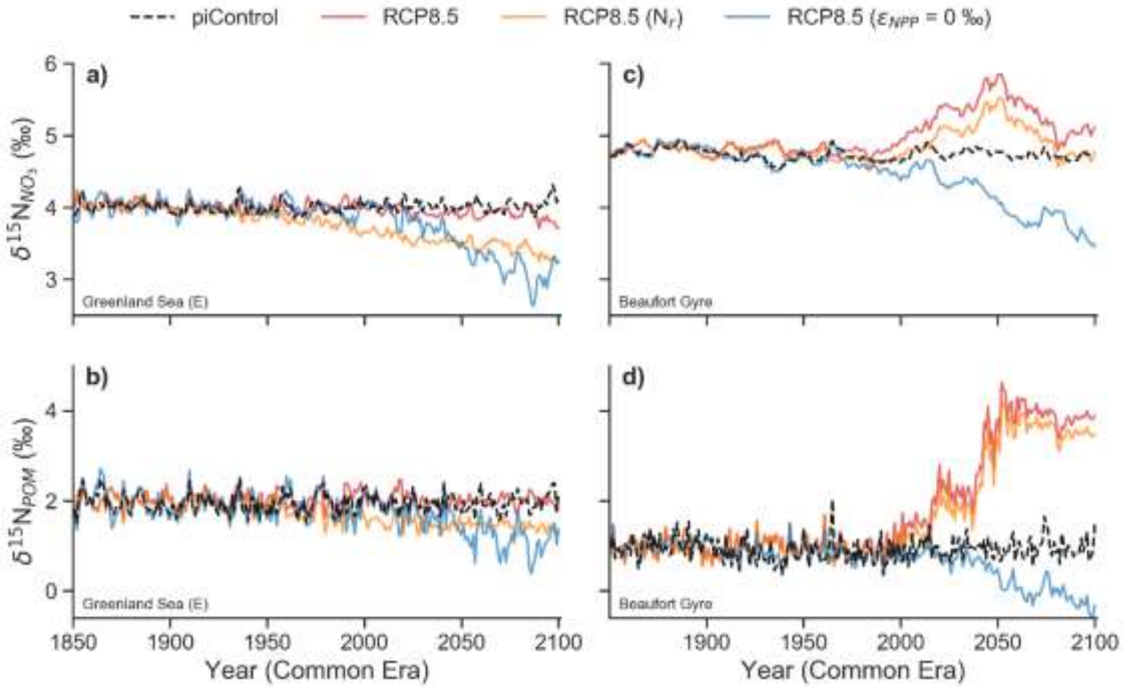

Figure S10. Inter-annual trends in nitrogen isotopes from 1850-2100 averaged over an Atlantic-influenced and Pacific-influenced area in the Arctic Ocean. Values are averaged over the upper 100 metres. a,c, Values of $\delta^{15}N$ (isoscape) of NO_3. b,d, Values of $\delta^{15}N$ (isoscape) of POM. Black line is the preindustrial control without anthropogenic emissions. The red line is the experiment with anthropogenic emissions. The orange line is the experiment with anthropogenic emissions and N_r deposition. The blue line is the experiment with anthropogenic emissions, without N_r deposition, and with no effect of primary production on $\delta^{15}N$. Regions are shown in Supplementary Figure 8.

References

Balmaseda, M. A., K. Mogensen, and A. T. Weaver. 2013. Evaluation of the ECMWF ocean reanalysis system ORAS4. *Quarterly Journal of the Royal Meteorological Society* 139: 1132–1161. doi:10.1002/qj.2063.

Koul, V., J.-E. Tesdal, M. Bersch, H. Hátún, S. Brune, L. Borchert, H. Haak, C. Schrum, et al. 2020. Unraveling the choice of the north Atlantic subpolar gyre index. *Scientific Reports* 10: 1005. doi:10.1038/s41598-020-57790-5.

Rafter, P. A., A. Bagnell, D. Marconi, and T. DeVries. 2019. Global trends in marine nitrate N isotopes from observations and a neural network-based climatology. *Biogeosciences* 16: 2617–2633. doi:10.5194/bg-16-2617-2019.

Reynolds, R. W., T. M. Smith, C. Liu, D. B. Chelton, K. S. Casey, and M. G. Schlax. 2007. Daily High-Resolution-Blended Analyses for Sea Surface Temperature. *Journal of Climate* 20: 5473–5496. doi:10.1175/2007JCLI1824.1.

Sigman, D. M., and F. Fripiat. 2019. Nitrogen Isotopes in the Ocean. In *Encyclopedia of Ocean Sciences*, 263–278. Elsevier. doi:10.1016/B978-0-12-409548-9.11605-7.

Taylor, K. E. 2001. Summarizing multiple aspects of model performance in a single diagram. *Journal of Geophysical Research* 106: 7183. doi:10.1029/2000JD900719.
